# Subitizing object parts reveals a second stage of individuation

**DOI:** 10.3758/s13423-020-01836-2

**Published:** 2020-11-17

**Authors:** Marlene Poncet, Ramakrishna Chakravarthi

**Affiliations:** 1 School of Psychology and Neuroscience, University of St Andrews, St Andrews, UK; 2 School of Psychology, University of Aberdeen, Aberdeen, UK

**Keywords:** Subitizing, Visual attention, Enumeration, Object recognition, Individuation

## Abstract

Humans can efficiently individuate a small number of objects. This *subitizing* ability is thought to be a consequence of limited attentional resources. However, how and what is selected during the individuation process remain outstanding questions. We investigated these in four experiments by examining if parts of objects are enumerated as efficiently as distinct objects in the presence and absence of distractor objects. We found that distractor presence reduced subitizing efficiency. Crucially, parts connected to multiple objects were enumerated less efficiently than independent objects or parts connected to a single object. These results argue against direct individuation of parts and show that objecthood plays a fundamental role in individuation. Objects are selected first and their components are selected in subsequent steps. This reveals that individuation operates sequentially over multiple levels.

**Supplementary Information:**

The online version contains supplementary material available at 10.3758/s13423-020-01836-2.

## Introduction

The enumeration of a small number of objects (up to three or four) is very fast and accurate. This operation, termed subitizing, contrasts with the error-prone enumeration at a glance of a larger number of objects, called estimation (Jevons, 1871; Kaufman et al., 1949; Mandler & Shebo, 1982). The difference in performance between subitizing and estimation seems to reflect two distinct systems, one dedicated to the precise representation of small individual objects and one to the representation of large, approximate numerical magnitudes (Feigenson et al., 2004).

Subitizing is a universal capacity present not only in humans (including babies) but also in animals across disparate genera such as monkeys, rats, parrots, pigeons, crows, bees and dolphins, highlighting its value to fitness (for reviews, see Feigenson et al., 2004; Gallistel & Gelman, 2000; Nieder, 2005). Subitizing has been argued to be an essential step in object recognition (Chakravarthi & Herbert, 2019; Pylyshyn, 2000; Xu & Chun, 2009) as it allows a few target objects to be individuated from the rest of a visual scene before being further processed and recognised. Accumulating evidence suggests that subitizing capacity is limited by attentional selection (Burr et al., 2010; Egeth et al., 2008; Mazza et al., 2013; Olivers & Watson, 2008; Pincham & Szűcs, 2012; Vetter et al., 2008). That is, attention can select and individuate only up to four items at a time. Given the importance of individuation in visual recognition and numerical cognition, it is essential to (a) examine what is subitized by the individuation mechanisms, and (b) determine where it takes place in the visual processing stream.

Early results suggested that objects are subitized only if they occupy distinct spatial locations (e.g. Pylyshyn, 2000; Trick & Pylyshyn, 1993); for example, concentric objects cannot be subitized. Further, in the presence of distracters, subitizing occurs for pop-out objects (e.g. O among X), but not for objects defined by a conjunction of features (e.g. vertical red bar among horizontal red and vertical green bars). That is, spatially separated and distinguishable objects are thought to be individuated by the subitizing mechanism.

However, far less attention has been paid to examining how components or parts of objects are enumerated. This is despite the fact that many if not most real-world objects have parts and individuating such parts is essential for everyday life (e.g., counting fingers, holding a kettle by its handle). Indeed, the dominant models of individuation (Mazza & Caramazza, 2015; Pylyshyn, 2000; Xu & Chun, 2009) are designed to account for enumerating distinct objects and do not make specific predictions for enumerating parts. Nevertheless, examining how parts of objects are subitized will shed light on both questions raised above: what is subitized and where does individuation occur.

Recently, a few studies have reported that subitizing parts of an object is as efficient as subitizing independent objects (Porter et al., 2016) and evoke the same neuronal signature (Poncet et al., 2016; Wurm et al., 2019). These results were taken to argue that components of an object occupying distinct locations, such as parts, can be individuated in parallel and that individuation is not bound to (distinct) objecthood. This would suggest that parts of an object are processed as if they were independent objects. These results are, however, seemingly at odds with other studies showing that tracking multiple objects is worse when objects are connected than when they are isolated (Scholl et al., 2001). Similarly, in the estimation range of enumeration, the total number of objects is underestimated when some are connected to each other (Franconeri et al., 2009; He et al., 2009, 2015). These findings might suggest that objecthood plays a role in individuation.

However, even though estimation is object-based in the sense that connectedness leads to underestimation, subitizing might not obviously be the same. Given the known differences between the two phenomena (Burr et al., 2010; Feigenson et al., 2004; Piazza et al., 2011), it is reasonable to expect that they might also differ in the effects of connectedness. Besides, it has been argued that numerosity estimates are derived from clustering processes (Im et al., 2016), which can be affected by connectedness. But such a process is unlikely to underlie subitizing. Instead, subitizing is argued to rely on attentional selection and this mechanism could potentially operate directly on parts of an object. In this case, objecthood or connectedness might not modulate subitizing.

This set of contradictory evidence raises the question of whether objecthood plays a central role in individuation and subitizing, which is fundamental in determining at which step individuation occurs during object recognition. To resolve it, we assessed if parts of multiple objects can be enumerated as efficiently as distinct objects or parts of a single object. Following convention, we operationalised efficiency as the time it takes to accurately enumerate each item (that is, the slope of reaction times plotted as a function of numerosity). If the individuation mechanism can directly select parts of objects just as it can select isolated objects, it should not matter whether the parts belong to one or to several objects. Similarly, performance should not be affected by the presence of distractor objects (without parts) in the display. In terms of efficiency, subitizing slopes should not be affected by these manipulations. On the other hand, if individuation is modulated by the distribution of parts among objects and/or the presence of distractors, subitizing efficiency would be impaired (individuation slopes would be steeper). This would suggest that objecthood plays a central role in subitizing. Importantly, knowing whether subitizing is affected by objecthood informs us about the sequence of visual processing steps. If our results show that subitizing is independent of objecthood, it would suggest that individuation takes place early, before visual features are integrated into separate objects. On the other hand, if subitizing is affected by objecthood, it would suggest that it relies on object-level representations.

## General methods

### Participants

Experiments 1, 2, 3 and 4 recruited 20, 20, 20 and 24 undergraduate participants, respectively. The subitizing effect is very robust and is observed in a large range of contexts and stimuli. Previous studies testing for differences in subitizing (e.g. Trick & Pylyshyn, 1993) have demonstrated large effect sizes. Hence, we expect a large effect (= 0.8 according to Cohen’s effect size index; Cohen, 1988). G*Power software (Faul et al., 2007) was used to compute the required sample size to detect such a difference between paired means (of subitizing efficiency) with α = .05, and power set to 0.8. The recommended sample size was 12; however, we opted to test at least 20 observers, given that some previous related studies (e.g. Porter et al., 2016; Trick & Pylyshyn, 1993) used 12–17 participants in their experiments.

Participants in this and subsequent experiments self-reported normal or corrected-to-normal vision and provided written informed consent. All experiments received the approval of the Psychology Ethics Committee, University of Aberdeen. Data for all studies are made available publicly available on Open Science Framework at https://osf.io/wv9hq/.

### Materials and stimuli

Participants were seated 57 cm from a CRT screen (27 cm screen width, 1,024 × 768 pixels resolution, 85-Hz refresh rate, 37.9 pixels per degree). The experiment was run using MATLAB (MathWorks, Natick, MA, USA) with Psychtoolbox extensions (Brainard, 1997; Kleiner et al., 2007). Stimuli consisted of one or more black circles with one to five black half diamonds (spikes) connected to them. Each experiment tested variants of this stimulus.

### Procedure

Each trial started with a grey fixation cross (size 0.25°) presented on a white background at the centre of the screen for 0.8–1.2 s. The stimulus, black circles and spikes, was then presented until participant’s response. Participants were asked to enumerate the number of spikes, from 1–5, as accurately and quickly as possible and report their answer using the number pad. As soon as a response was made, the stimulus disappeared for 0.3 s and a 750-Hz note was played for 100 ms if the response was incorrect. The fixation cross remained visible for the entire trial. To make sure that participants were able to use the number pad accurately and rapidly, they were trained before the experiment to report as quickly and as accurately as possible a digit presented in the lower half of the screen using the number pad. Each digit (1–5) was repeated 10 times during this training session.

### Analysis

We obtained the error rate and the median reaction time (RT) on correct trials for each spike numerosity and condition. With a maximum of five objects, we can assess subitizing performance while avoiding performance distorted by end effects (Trick & Pylyshyn, 1993). To assess the efficiency of subitizing, we computed the RT slope in the subitizing range using a linear fit. The subitizing range usually spans up to three or four objects, with considerable individual differences. To avoid including numerosities outside the subitizing range, we computed RT slopes between numerosities 1 and 3. These slopes were calculated for each participant and each condition separately. Appropriate statistics (pairwise comparisons or repeated-measure ANOVAs) were then applied to the RT slopes. All mean values are reported with their 95% within-subject confidence intervals calculated using the Cousineau-Morey method (Cousineau, 2005; Morey, 2008). All graphs were plotted in MATLAB using the gramm toolbox (Morel, 2018).

## Experiment 1: Replication and extension of Porter et al. (2016)

We first replicated previous results testing the enumeration of parts of an object (e.g. Porter et al., 2016) with the procedure and stimuli that we planned to use in subsequent experiments.

### Method

#### Stimuli

**Fig. 1:**
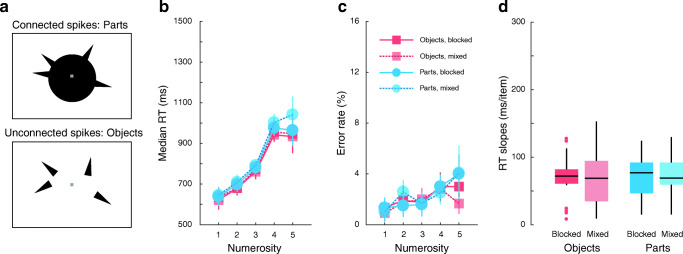
Stimuli and results of Experiment 1. (**a**) Illustration of the stimuli used in Experiment 1. The spikes were either connected to an object (parts enumeration) or unconnected (objects enumeration). In all experiments the stimulus was presented until participants reported the number of spikes (1 to 5). Median reaction times (RTs; **b**) and error rates (**c**) for parts (blue circles) and object enumeration (red squares) as a function of numerosity in blocked (saturated symbols) and mixed (faded symbols) conditions. Error bars represent within-subject 95% confidence interval. (**d**) Subitizing efficiency (RT slopes calculated between one and three items) for each enumeration type (part or object) and block type (blocked or mixed)

Between one and five black half diamonds (spikes) were placed at the edge of a circle of diameter 6° of visual angle. The latter circular space was occupied by a filled black circle in half of the trials and unfilled black circle in the other half (Fig. 1a). The spikes were distributed according to an algorithm. First, one spike was placed at a randomly chosen location along the circumference of the circle. Eleven other locations were then identified at the circle’s edge, each 30 polar degrees apart. The remaining number of spikes were randomly allocated to these locations. The minimum separation between spikes was chosen to avoid crowding and overlap masking. The width of the spikes was randomised to be between 0.2° and 0.5° of visual angle. The height of the spikes (the distance between the circle border and the tip of the spike) was 2.5 times its width. The long axis of the spikes was perpendicular to the circumference of the circle. A small jitter between ±3 polar degrees was, however, added to each spike’s orientation. A further jitter varying between either -15 and -30 or between +15 and +30 polar degrees was added to each spike when a physical circle was not presented. This was done to prevent the perception of illusory contours that might be constructed from the bases of the spikes.

#### Design

Participants were asked to enumerate the spikes that were presented irrespective of the presence of a circle. Spikes connected to the circle are considered parts and those without such a connection are considered objects. Previous studies used blocked designs (all trials within a block tested part enumeration or object enumeration), which can potentially introduce expectation-related, perceptual or other strategies as confounds. To examine whether this was the case, we tested both blocked and mixed designs: participants enumerated spikes that were objects or parts in separate blocks (blocked condition) or within the same block (mixed condition). Each block consisted of 100 trials, corresponding to 20 repetitions per spike numerosity. Participants completed 12 blocks in total: six mixed blocks, three object-enumeration blocks and three part-enumeration blocks. In total, there were 60 trials per condition.

### Results

As expected, median RT (Fig. 1b) and error rates (Fig. 1c) increased with the number of spikes while accuracy remained high for numerosities 1–3 (around 98%). There was no effect of enumeration type (F(1,19) = 0.02, p = 0.89, _p_η^2^ = 0.001), of block design (F(1,19) = 0.58, p = 0.45, _p_η^2^ = 0.03), nor was there an interaction between these two factors (F(1,19) = 0.18, p = 0.68, _p_η^2^ = 0.009) on subitizing RT slopes (Fig. 1d). These findings replicate previous reports that subitizing parts and objects are equally efficient. It further shows that this is the case regardless of the design (blocked or mixed), indicating that participants’ expectation did not influence their subitizing ability.

## Experiment 2: Subitizing parts distributed among multiple objects

This experiment was designed to test the role of objecthood in enumerating parts by examining whether parts connected to multiple objects can be subitized as efficiently as unconnected parts or parts connected to a single object.

### Method

#### Stimulus

**Fig. 2:**
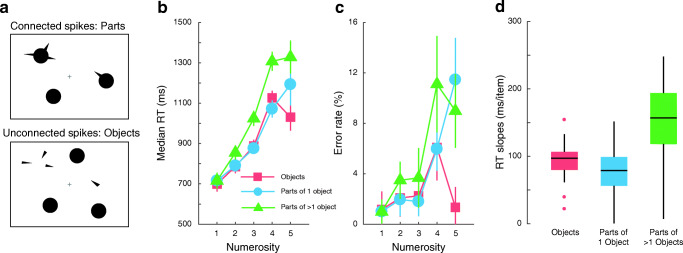
Stimuli and results of Experiment 2. (**a**) Participants enumerated the number of spikes unconnected or connected to one or more circles. Median RT (**b**), error rates (**c**) and subitizing efficiency (**d**) for enumerating objects (or unconnected spikes: red squares), parts of one object (blue circles) and parts of multiple objects (green triangles). Note that when a single spike is connected to a circle, the stimulus is identical for the two kinds of parts conditions: ‘parts of one object’ and ‘parts of multiple objects’

The stimulus consisted of 1–5 circles of diameter 0.8° along with 1–5 spikes of width 0.24° and height 0.6° (Fig. 2a). The spikes were either connected to the circles (‘parts’) or unconnected (‘objects’). Circles were present in both conditions. This means that the number of occupied pixels remained the same in both conditions, unlike in previous studies (Porter et al., 2016; Wurm et al., 2019) or in our replication (Experiment 1). All combinations of the number of circles (1–5), the number of spikes (1–5), and stimulus type (parts and objects) were tested. On each trial, the specified number of circles were assigned to random cells within an invisible 4 × 4 grid centred on the fixation cross. Adjacent cells in this grid were 2° apart. A subset of the available circles was chosen, and the spikes were randomly assigned to these circles. In trials where the spikes were distinct objects, the same procedure was applied except that the circles were instead drawn in randomly chosen cells of the grid that were previously unselected (and hence unoccupied by the spikes). Further, the orientations of some of the spikes in the object condition were ‘flipped’ by 180° to prevent the formation of illusory contours. A small orientation jitter of ± 3 polar degrees was added to all spikes. If more than one spike was allocated to a circle or cell, they were at least 60 polar degrees apart to avoid crowding and overlap masking. A positional jitter of ± 3 pixels in both horizontal and vertical directions was added to each circle.

#### Design

The experiment consisted of six blocks of 100 trials each. Each block included 50 trials where spikes were presented unconnected to any of the presented circles (objects condition) and 50 trials where they were connected to one or several circles (parts conditions). Among the latter, spikes were connected to one circle (parts of a single object) in approximately half the trials, and in the other half to more than one circle (parts of multiple objects). The order of trial condition was randomised within each block. The results were analysed by comparing the subitizing slopes among the three conditions where the spikes were: (1) disconnected, separate objects, (2) connected to one object and (3) connected to multiple objects.

### Results

Participants’ performance worsened with increasing number of spikes (Fig. 2b and c). Within the subitizing range, error rates remained very low, around 2% when spikes were disconnected or connected to one object and around 3.5% when they were connected to more than one object. Enumerating parts connected to multiple objects was slow and inefficient with subitizing slopes of 154 ± 16 ms/item (Fig. 2d and Table 1). It was substantially steeper than (a) subitizing separate objects by 60 ± 19 ms/item (t(19) = 6.52, p < 0.001, Cohen’s d = 1.46; Bonferroni-corrected for three comparisons) and (b) subitizing parts of a single object by 74 ± 21 ms/item (t(19) = 7.45, p < 0.001, Cohen’s d = 1.66). Thus, individuating a small number of parts located on multiple objects is highly inefficient, substantially beyond the range (40–100 ms/item) considered to characterise subitizing (Trick & Pylyshyn, 1994).

In addition, our results show that subitizing objects was slightly less efficient than subitizing parts connected to one object by 14 ± 12 ms/item (t(19) = 2.46, p = 0.07, Cohen’s d = 0.55). This result is surprising as one would have expected comparable slopes in both conditions, as found by Porter et al. (2016) or in Experiment 1. One potential explanation is the presence of circles with no spikes (distractors) in this experiment. This particularly affects the object condition as the total number of on-screen objects (circles and separate spikes) becomes larger than 5 whereas it remains between 1 and 5 in the parts condition. Thus, the higher number of distractors in the object condition could result in less efficient subitizing slopes.

## Experiment 3: Subitizing parts of one or two objects with or without the presence of distractors

Experiment 3 systematically assessed, in a 2 × 2 design, the effect of distributing parts among multiple objects and the role of distractor presence. Here, parts were connected to either one or to two objects. In addition, these objects-with-parts could be presented with distractor objects with no parts while keeping the total number of objects on the screen constant.

### Method

#### Stimulus

The stimulus consisted of one to four circles of diameter 2° and one to five spikes of size 0.24° × 0.6°. The circles were randomly assigned to cells within an invisible 4 × 4 grid with an inter-cell spacing of 4°.

#### Design

**Fig. 3:**
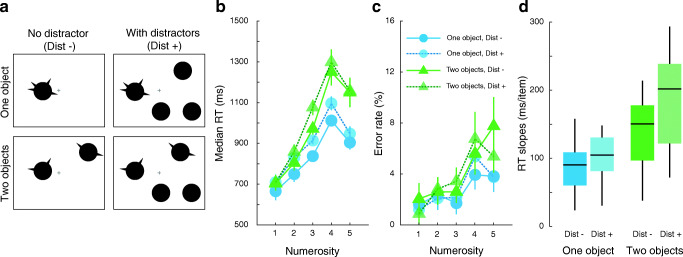
(**a**) In Experiment 3, participants enumerated spikes connected to one (blue circles) or two (green triangles) circles in the presence (faded symbols; Dist +) or absence (saturated symbols; Dist -) of distractors. Effect of the number of objects with parts and the presence of distractor on participants’ median reaction time (RT; **b**), error rates (**c**) and subitizing efficiency (**d**)

Spikes were always connected to one (one object condition) or to two (two objects condition) circles. In addition, distractor circles with no spikes were presented on half the trials. When distractor circles were present, the total number of objects on the screen was always equal to four. That is, in the one object condition with distractors, participants were presented with one circle with spike(s) together with three circles without spikes. In the two objects condition with distractors, participants were presented with two circles with spike(s) together with two circles without spikes (Fig. 3a). The experimental session consisted of 15 blocks of 120 trials. In total, each spike numerosity (1–5) was tested with 90 trials in each condition.

### Results

Participants’ accuracy decreased and reaction times increased with the number of spikes in all conditions (Fig. 3b and c). Accuracy nevertheless remained high with error rates of around 2% within the subitizing range. Subitizing slopes (Fig. 3d and Table 1) were more efficient, by around 33 ± 7 ms/item, when the spikes were connected to one object compared to when they were connected to two objects (F(1,19) = 64.46, p < 0.001, _p_η^2^ = 0.77). The presence of distractors also increased subitizing slopes by 65±17 ms/item (F(1,19) = 93.47, p < 0.001, _p_η^2^ = 0.83). An interaction between number of objects and distractor presence was also observed (F(1,19) = 26.87, p < 0.001, _p_η^2^ = 0.59). This interaction revealed that the effect of distractor presence was larger when participants had to enumerate spikes on two objects compared to when they had to enumerate spikes on only one object (t(19) = 5.18, p < 0.001, Cohen’s d = 1.16). Specifically, the presence of distractors increased subitizing slopes by around 16 ± 11 ms/item in the one-object with parts condition (86 ms/item vs. 103 ms/item) compared to 50 ± 9 ms/item in the two-objects with parts condition (134 ms/item vs. 184 ms/item). That is, the effect of distractors was threefold higher when parts were distributed among two objects than when they were on one object. Note that enumerating parts of two objects with or without distractors was highly inefficient, and beyond the 40- to 100-ms/item subitizing range. Thus, both distributing parts among more than one object and the presence of distractors decreased subitizing performance. This strongly indicates that subitizing is affected by objecthood.

## Experiment 4: Role of distance and objecthood on subitizing parts

Experiments 2 and 3 provide evidence that subitizing parts of multiple objects is less efficient than subitizing parts of a single object. One possible account for this result is that the average distance between parts is shorter when all parts are connected to one object than when they are distributed among multiple objects. To test if this can explain our results, in Experiment 4a, we examined the effect of distance for enumerating parts distributed over two objects. In Experiment 4b, we fixed the distance between parts and manipulated objecthood by distributing parts over one or two objects.

### Method

#### Stimulus

##### Experiment 4a

**Fig. 4:**
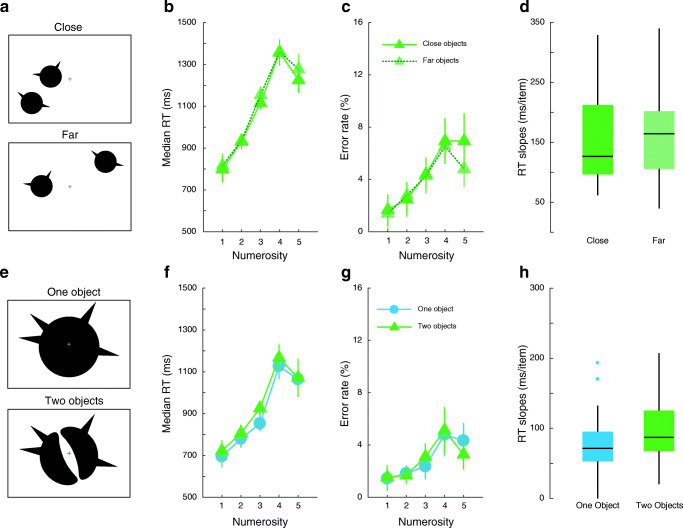
Illustration of stimuli and participants’ performance in Experiment 4a (**a**, top row) and 4b (**e**, bottom row). Median reaction time (RT; **b**), error rates (**c**) and subitizing efficiency (**d**) for enumerating parts located on two objects that were either close (saturated symbols) or far (faded symbols) from each other. Median RT (**f**), error rates (**g**), and subitizing efficiency (**h**) depending on whether the spikes were located on one (blue circles) or two objects (green triangles) while controlling for distance

One to five spikes of size 0.2° × 0.5° were connected to two circles of diameter 2° (Fig. 4a). The two circles were separated by either 3.1° (close condition) or 9.3° (far condition, three times further away). The first circle was presented at a random location between 1.8° and 8.5° from the centre of the screen. To ensure that the two conditions did not differ in eccentricity, we then placed the other circle at the appropriate distance (close or far) as long as it was between 1.8° and 8.5° from fixation.

##### Experiment 4b

One to five spikes were connected to one circle or to two ‘bean’-shaped objects placed symmetrically around fixation (Fig. 4e). First, we constructed the stimulus for the one-object condition. For that, we divided the circle into four quadrants. Three equidistant locations were identified at the edge of the circle in each quadrant. The first spike was placed randomly in one of these 12 locations. When there were two or more spikes, the second spike was presented in one of the two quadrants across the vertical meridian. Further spikes (as required) were randomly distributed among the remaining ten locations. On each trial the diameter of the circle was chosen to be 4°, 8° or 12°. All sizes were tested an equal number of times. The spike size was correspondingly scaled to be 1°, 2° or 3° in height. The whole circle including the part(s) was then rotated to a random orientation on each trial. For each choice of spike location, circle size and orientation, we created an equivalent trial where the circle was replaced by two symmetrical bean-like objects of the same external curvature as the circle, thus equating distance between parts in the two conditions.

#### Design

The two experiments (4a and 4b) were conducted in the same session with the same participants. The order of experiment was counterbalanced across participants. There were 1,440 trials in total, corresponding to 72 trials per condition. The order of trials was randomised within each experiment.

### Results

#### Experiment 4a

Enumeration performance worsened with increasing number of spikes (Fig. 4b and C). Accuracy remained high with an error rate of 3% within the subitizing range. It can be noted however that in both close and far conditions, error rate increased with numerosity from 1 to 3 whereas it had remained relatively flat in previous experiments. RT enumeration slopes were inefficient (158 ± 16 ms/item in the near condition and 169 ± 16 ms/item in the far condition, Table 1) in line with the findings of Experiments 1 and 2. Importantly, despite an approximately threefold change in average distance between parts, RT slopes did not differ with distance (Fig. 4d; t(23) = 0.92, p = 0.37, Cohen’s d = 0.19). Therefore, the average distance between parts cannot explain the previous results.

**Table 1:** Average reaction time (RT) slopes in ms/item for enumerating between one and three items in all experiments

Experiment	Condition	RT slope	95% CI
1	Distinct objects, blocked design	70	10
	Distinct objects, mixed design	69	6
	Parts, blocked design	72	7
	Parts, mixed design	73	6
2	Objects (with distractors)	94	10
	Parts on 1 object (with distractors)	80	11
	Parts on more than 1 object (with distractors)	154	16
3	Parts on 1 object, no distractor	86	13
	Parts on 1 object, 3 distractors	103	10
	Parts on 2 objects, no distractor	134	8
	Parts on 2 objects, 2 distractors	184	13
4a	Parts on 2 close objects	158	16
	Parts on 2 far objects	169	16
4b	Parts on 1 object	79	9
	Parts on 2 objects (controlling for distance)	101	9

#### Experiment 4b

Participants were very good at enumerating spikes connected to one or two objects with error rates below 3% in the subitizing range (Fig. 4f). RTs increased with numerosity (Fig. 4g). Subitizing slopes were steeper when the parts were located on two objects (101 ± 9 ms/item) than on one object (79 ± 9 ms/item) (Fig. 4h and Table 1, t(23) = 3.81, p < 0.001, Cohen’s d = 0.78). Thus, although the distance between parts was equated, subitizing parts of two objects was less efficient than subitizing parts belonging to one object. Relatively speaking, subitizing slopes in Experiment 4b were efficient, even when parts were distributed among two objects. This might be because the parts were quite large relative to the objects, or that the position of the objects were fixed, known and close to the fovea (unlike in Experiments 2 and 3), or that the two bean-shaped objects were not as separable compared to other experiments since they were very close and symmetrically placed; that is, they might have been weakly perceived as a single object.

We tested the effect of relative part size and potential object binding with a different set of stimuli inspired by the classic study demonstrating object-based attention (Egly et al., 1994). The distance between parts was controlled but the two objects with parts were unlikely to appear bound to each other (Supplementary Experiment 1, Online Supplementary Material). The results were comparable to that in Experiment 4b. Overall, in both experiments, the number of objects strongly modulated subitizing slopes (a 25–30% increase) suggesting that objecthood is a crucial factor determining subitizing efficiency.

The specific configuration of the stimuli in Experiment 4b might also create a prominent gap between the two objects, which itself might attract attention, thus reducing the resources available for extracting parts. However, note that the control experiment (Supplementary Experiment 1, Online Supplementary Material) demonstrated an effect of objecthood despite not having a prominent gap, which suggests that the gap might not have played a substantial role in modulating subitizing efficiency. A second possibility is that if the two objects appear bound, the perceived topology changes (the two objects are perceived as a single object with a hole in the middle). This difference in topology might underlie differences in subitizing efficiency, since it is also known to affect other visual capabilities (Chen, 2005). We tested both of these confounds by asking participants to enumerate parts of an object with or without a hole in the centre (Supplementary Experiment 2, Online Supplementary Material). We found no difference in subitizing slopes indicating that attention being attracted by the gap or a change in topology cannot account for our results.

In sum, the replication of the results with a different set of stimuli (Supplementary Experiment 1, Online Supplementary Material) along with no observed differences in subitizing efficiency in the presence of changes in distance (4a) or topology (Supplementary Experiment 2, Online Supplementary Material) suggest that subitizing is object-based.

## General discussion

The purpose of this study was to test if individuation and subitizing rely on object-based mechanisms by examining if parts of multiple objects could be efficiently individuated. Since we routinely need to individuate parts for carrying out everyday activities, from drinking tea to driving, it is important to uncover the processing pipeline for individuating objects and parts. We found that parts of multiple objects could not be enumerated as efficiently as separate objects (Experiment 2) or as parts of one object (Experiments 2, 3, and 4b, and Supplementary Experiment 1). Further, the presence of distractors (objects without parts) worsened subitizing efficiency (Experiments 2 and 3). These findings cannot be attributed to a possible distance confound between conditions (Experiments 4a and 4b, Supplementary Experiment 1), to topological differences or attentional diversion to irrelevant aspects of the stimulus (Supplementary Experiment 2). We argue that these results demonstrate that subitizing relies on object-based mechanisms. They further throw fresh light on the mechanism underlying individuation.

One of the most influential models of subitizing and individuation is the FINST theory proposed by Pylyshyn (2000). This model posits that after visual features are independently detected in parallel, they are grouped according to Gestalt processes. Up to four FINSTs are pre-attentively assigned to such clusters which allows them to be individuated and subitized. These clusters are subsequently selected by attention for feature integration and identification. However, accumulating evidence suggests that attention is necessary for individuation and subitizing (Burr et al., 2010; Egeth et al., 2008; Mazza et al., 2013; Olivers & Watson, 2008; Pincham & Szűcs, 2012; Vetter et al., 2008). These results prompted an update to the sequence of processing stages such that attentional selection was considered the limiting factor that allows up to four loosely clustered features to be individuated (Mazza & Caramazza, 2015; Xu & Chun, 2009). Further, the recent finding that parts of an object can also be efficiently individuated (Poncet et al., 2016; Porter et al., 2016; Wurm et al., 2019; Experiment 1) argued for an extension of the model that attention need not select only objects; it can also select parts of an object. That is, even though the parts are tightly bound to an object by the Gestalt principle of connectivity, the selection mechanism can isolate up to four parts, perhaps before the stage they are bound to the object.

However, our findings throw a wrench in this sequence of processing steps. If parts could be selected before integration into the objects, or in parallel, then their enumeration should be efficient even if they belonged to different objects. Further, their extraction should not be substantially affected by the presence of part-less distractors. This is not what we find. Subitizing efficiency is affected both by the number of objects over which parts are distributed and the presence of distractors. This indicates that objecthood plays an important role in the sequence of events. To account for these results and previous findings, we propose that individuation is a two-step process.

Consistent with previous models, features are initially detected and then grouped according to Gestalt principles. In the first individuation step, attention can select up to four target clusters that can be individuated and enumerated (Trick & Enns, 1997; Watson et al., 2005). These clusters might be unbound, albeit grouped, features or fully bound objects. Our proposal is agnostic as to their precise state. Crucially, this step selects *only clusters* and cannot operate over parts or other components of an object. Only then in a second step can the subitizing mechanism access parts or components of objects.

If parts of an object have to be enumerated, in the special case where no other objects are present, parts can be subitized as efficiently as distinct objects (Poncet et al., 2016; Porter et al., 2016; Wurm et al., 2019; see also Experiment 1). This is because the single object with parts is initially individuated at no cost by attention. It therefore appears *as if* the same individuation mechanism supports subitizing parts of a single object and distinct objects. However, in the presence of distractors, attention has to first select the clusters or objects with parts among other objects and then their parts can be enumerated. Hence, adding distractors reduces subitizing efficiency (Experiments 2 and 3).

It might be argued that this effect of distractors need not indicate an object-based mechanism but instead reflects a filtering process that works on some featural difference (say size and shape) between targets and distractors. If this were the case, the visual system should be able to use the featural difference between parts and the other items in the display to filter the latter. However, it seems unable to do so and instead selects whole objects first (circles *with* parts) in the presence of distractors (or filters out just the distractor objects). In addition, there is indirect evidence that only objects, or feature clusters, can be selected by subitizing mechanisms. Although featural differences are constant, enumerating the number of colours of clustered set of coloured dots is more efficient than that of a intermixed set (Watson et al., 2005). Similarly, access to components is impaired when integrated within a larger Gestalt context (Pomerantz et al., 1977) or a recognisable object (Poljac et al., 2012). Hence, even if a featural difference among objects (size, colour, etc.) can invoke a filtering mechanism, what is filtered/selected operates on the level of object representations (only objects are filtered).

Our findings show that, even in the absence of distractors, subitizing parts distributed among multiple objects is less efficient than when they are present on a single object. This is the case even when the distance between parts is equated (Experiment 4, Supplementary Experiment 1). One possibility is that parts of one object are extracted followed by parts of another. Because it takes time for attention to move between objects, subitizing is less efficient. Alternatively, it is possible that once the target objects are selected, parts are extracted in parallel for all objects but then the numerosities of the parts from each object need to be added. This step of addition would slow down the enumeration process. Our experiments do not distinguish between these accounts. However, they all argue that individuation mechanisms operate at the level of grouped features (objects or clusters) and a second step of selection is required for parts to be individuated.

In conclusion, this study provides evidence that subitizing is not an object-independent mechanism. Instead, we propose that individuation takes place in two steps: first, clusters of grouped features (or objects) are individuated, and subsequently their parts. Further, each of these individuation steps is limited by attentional resources.

## Supplementary Information

ESM 1 (DOCX 200 kb)
