# Supplementary material for: Subitizing object parts reveals a second stage of individuation
Source: Psychon Bull Rev. 2020 Nov 17;28(2):476–86. doi: 10.3758/s13423-020-01836-2 (PMC8062358; doi:10.3758/s13423-020-01836-2)
Supplement: Supplementary file 1 — (DOCX 200 kb) [file 13423_2020_1836_MOESM1_ESM.docx]

# Supplementary Experiment 1

The results of Experiment 4b indicate that subitizing parts of two objects is less efficient than subitizing parts of one object, even when the distance between parts is controlled for. However, one possible confound in the stimulus used in that experiment is that the two objects were close and symmetrically arranged, which might have encouraged participants to view it as a single object. We note that, despite this, the subitizing slopes were higher in the two objects condition. Nevertheless, following the advice of a reviewer, we designed a new set of stimuli that would preclude the possibility of perceptual binding between objects, while controlling for the distance between parts. This stimulus was inspired by that used in a series of studies that tested object-based attention (Egly et al., 1994). This experiment has two main purposes. First, it allows us to test the generalisability of our findings to a completely different set of stimuli, particularly one where the parts are much smaller relative to the objects than in the other experiments. Second, it tests whether the apparent perceptual binding between the two objects in Experiment 4b might have modulated the outcomes.

## Method

Nine observers (including the two authors) took part in this experiment. The experiment was conducted in an unorthodox manner^[[1]](#footnote-1)^. The experimental program, written in MATLAB and Psychtoolbox, was emailed to the volunteers who ran it on their personal computers. Hence, the properties of the monitor and computer would be different for each participant. The participants were encouraged to sit at reading distance from the screen (about 40 cm), but this cannot be ensured. Taken together, the sizes of the stimuli were uncontrolled and cannot be precisely reported. This might be considered an issue in perceptual studies, but it might also be useful, at least to some extent, to gauge the generalisability of the effect across different settings. All sizes of the stimuli were set as a percentage of the screen width in pixels, so as to ensure similar coverage across all screen sizes.

One to five ‘pegs’ (of height 0.8, 1.6 or 2.4% of screen width and width that was one-third of the height) were connected to either one long rectangle (one object condition) or two squares (two objects condition). The squares and rectangles were unfilled quadrilaterals with a black border of thickness equal to one-third of the height of the pegs, and hence with the same width as the pegs (Figure S1A). The sides of the square had a length (*m*) of 4, 8 or 12% of the screen width. The dimensions of the rectangle were *m* x 3*m*. The two squares were separated (edge-to-edge) by a distance equal to *m*. Thus, the two squares would be at the same locations as the ends of the rectangle. The number of occupied pixels would also be the same. The two squares or the rectangle would be presented either to the left, right, top or bottom of fixation. The rectangle was centred at a distance *m* from the fixation in one of the four cardinal directions. The squares were centred at a distance *m*$\surd2$ from fixation (top left, bottom left, bottom right or top right visual quadrant). The orientation of the rectangle was determined by its location, such that its long side always faced the fixation. The size of the pegs scaled with the size of the objects. The part/object size ratio was substantially smaller than in Experiment 4b.

In the two objects condition, three equidistant locations were identified on each of the two ‘outer’ sides of each square (sides furthest from fixation). One peg was placed at one of these twelve locations. If the trial numerosity was greater than one, the second peg was placed on one of the six locations on the other square. The remaining pegs (for numerosities higher than two) were placed randomly in the remaining 10 locations. A small jitter of ±3 pixels was added to the location of the pegs.

The two squares were replaced with a rectangle in the one object condition. Further, in both these conditions two additional squares were presented as distractors. This was done to ensure parity across both conditions: if there were no distractors in the stimulus, in numerosity one trials there would nevertheless be one distractor in the two objects condition but not in the one object condition.

The procedure was the same as in Experiment 4b. Each numerosity (1-5) and number of objects with parts (one or two) was tested with 80 trials, leading to a total of 800 trials per participant.


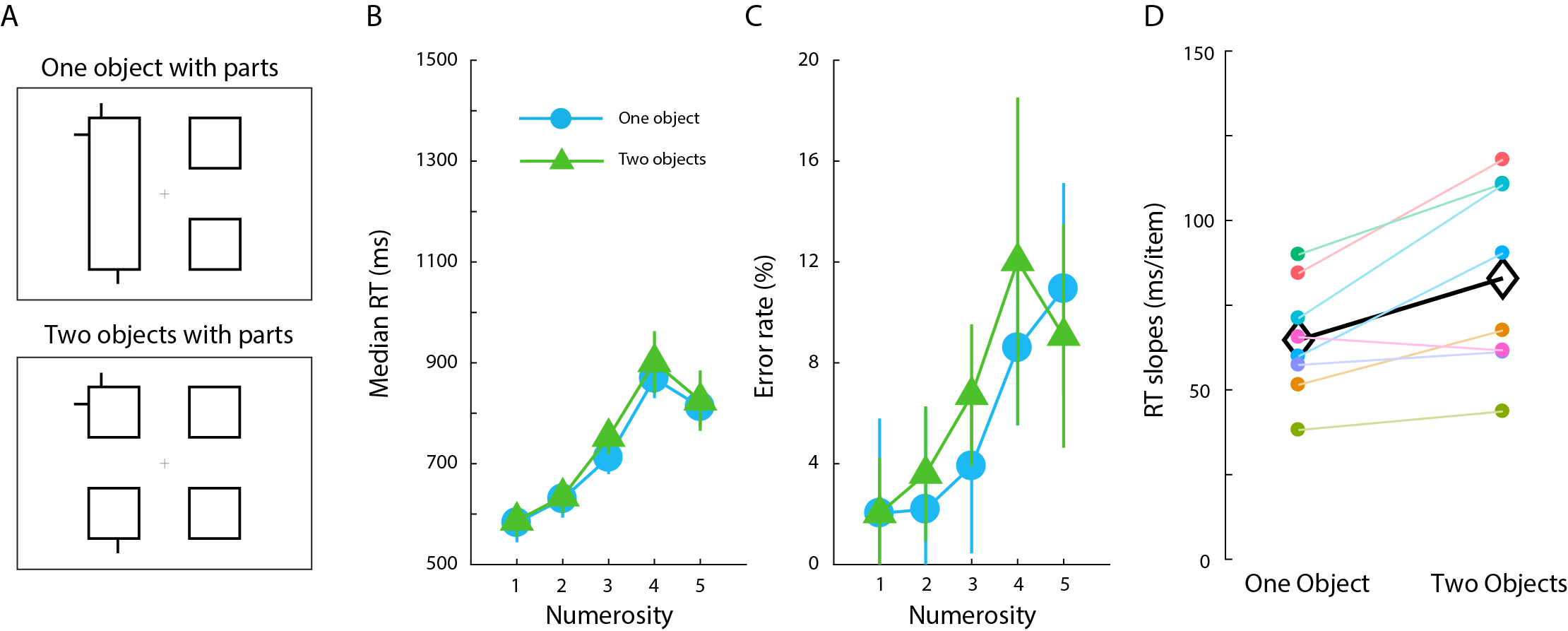


Figure S1. Stimuli and results of Supplementary Experiment 1. **A.** Pegs were connected to one or two squares. Participants’ median RT (**B**) and error rates (**C**) for enumerating pegs distributed on one (blue circles) or two (green triangles) objects. (**D**) Subitizing efficiency (RT slopes calculated between 1 and 3 items) for enumerating parts on one or two objects. Each colored circle represents one participant. Black diamonds represent the mean across all participants. All participants, but one, demonstrate a worsening of efficiency when enumerating parts distributed on two objects. Error bars represent within-subject 95% confidence intervals.

## Results

We excluded one participant’s data from analysis (final n=8) because the stimulus was rendered incorrectly on their laptop. Although participants were accurate at enumerating 1-3 pegs in the subitizing range (Fig. S1C), error rates were higher in the two objects condition (4.4%±1.5%) than in the one object condition (2.7%±1.5%; this difference did not reach significance: t(7) = 1.52, p = 0.17, Cohen’s d = 0.54; but we need to keep in mind that power might have been low for such tests). Importantly, as in Experiment 4b, subitizing slopes were steeper when parts were located on two objects (83±9 ms/item) than on one object (65±9 ms/item) (Fig. S1D, t(8)=3.3, p=0.013, Cohen’s d=1.17). Crucially, seven among eight observers had steeper subitizing slopes in the two objects condition than in the one object condition, indicating that it is a robust effect. The slope in the two objects condition was higher than that in the one object condition by around 28%, about the same as was seen in the main Experiment 4b. These findings, in conjunction with the results of Experiment 4b, suggest that neither the perceived binding between the two objects in the two objects condition nor the size of the parts relative to the object can explain lower subitizing efficiency for parts distributed on two objects compared to one object.

One might, therefore, wonder why subitizing slopes are relatively efficient here, despite the presence of distractors, compared to previous experiments (cf. Experiment 3). We think that two factors might have affected subitizing efficiency in this experiment. First, the positions of the pegs were relatively predictable to the participants: they were always located on the eight segments/sides that were away from fixation. Hence one could (pre)allocate attention to these four corners. Second, unlike in Experiment 3, there could be no intervening stimulus between two objects with parts (i.e. there was no need to split attention between the two objects with parts). These two factors, predictability of the peg locations and the absence of intervening stimuli, affect how attention is allocated to the stimuli. Since subitizing relies on attentional resources, they will therefore affect subitizing efficiency.

# Supplementary Experiment 2

The results of Experiment 4b can also be explained as a consequence of a change in (perceived) topology. In the two objects condition, the close and symmetrical placement of the objects might have led participants to view it as a single object with a hole in it, which would change the topology of the stimulus. It is known that topology affects various visual processes, including numerosity estimation (Chen, 2005; He et al., 2015; Kluth & Zetzsche, 2016). Thus, the modulation of subitizing efficiency in Experiment 4b could be attributable to a topological difference and not to an object-based mechanism. Alternatively, the presence of a gap between the two objects might have attracted attention to it. This drawing of attentional resources to the gap could reduce the efficiency of subitizing in the two objects condition, again explaining the results not in terms of an object-based mechanism. This experiment was designed to test these alternatives.


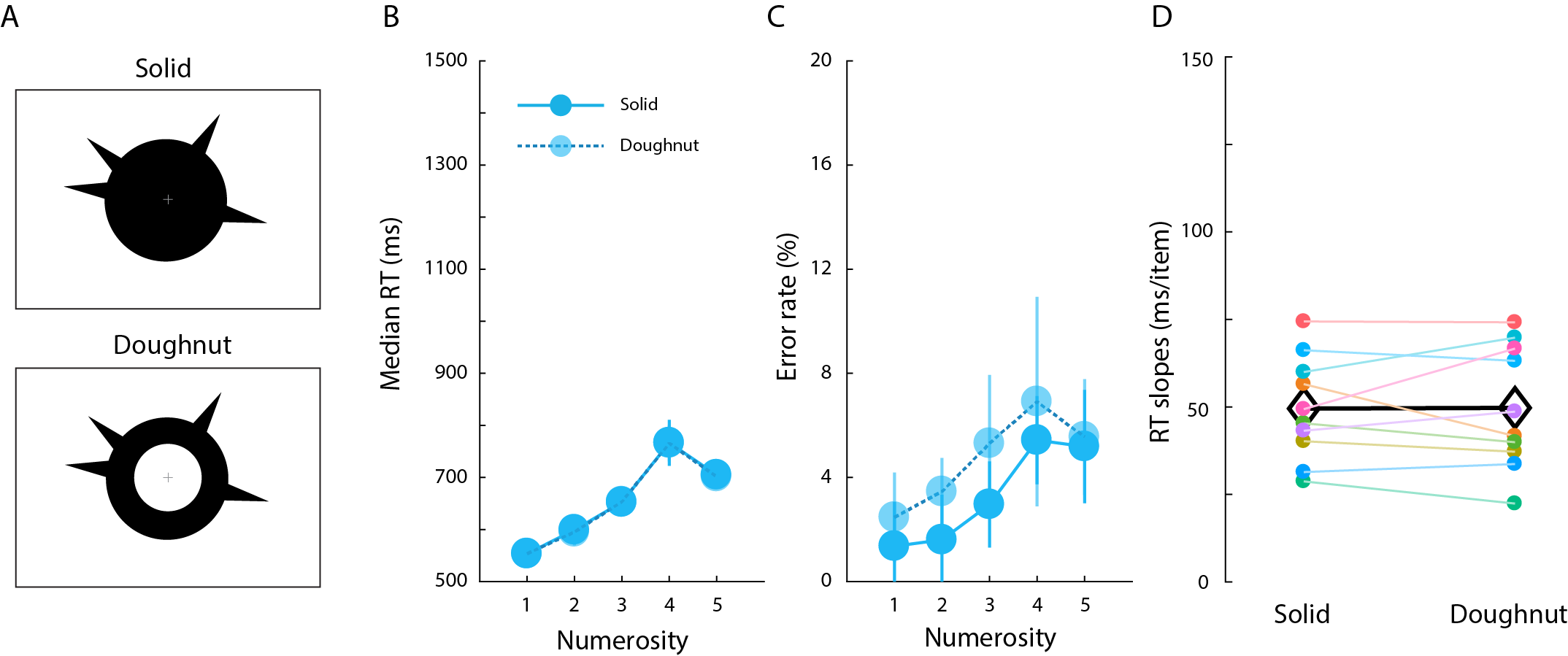
 Figure S2. Stimuli and results of Supplementary Experiment 2. **A.** Spikes were connected to a solid circle or a doughnut. Participants’ median RT (**B**) and error rates (**C**) for enumerating spikes distributed on a solid circle (dark blue circles) or a doughnut (light blue circles). (**D**) Subitizing efficiency. Each colored circle represents one participant. Black diamonds represent the mean. Error bars represent within-subject 95% confidence intervals.

## Method

Ten observers (including the two authors) took part in this experiment. The materials were as in Supplementary Experiment 1. One to five spikes of height 5, 7.5 or 10% of screen width were connected to a circle of diameter 10, 15 or 20% of the screen width, respectively (Figure S2A). In half the trials, the circle was entirely filled and in the other half, a smaller circular region with a diameter of 8, 12 or 16% of screen width was left unfilled (and had the background colour). Thus, the black circle appeared, instead, as a doughnut. This manipulation adds a hole (changes topology) and should also elicit the putative draw of attentional resources. The arrangement of spikes and the procedure was the same as Experiment 1. Each numerosity and topology condition combination was tested with 80 trials (a total of 800 trials).

## Results

Participants were very accurate at enumerating in the subitizing range (Fig. S2C); however, they made slightly more errors in the doughnut condition (3.7%±1%) than in the solid circle condition (2%±1%; t(9)=2.7, p = 0.025, Cohen’s d = 0.84). Crucially, subitizing slopes in the solid circle condition (50±5 ms/item) was the same as in the doughnut condition (50±5 ms/item; Fig. S2D; t(9)=0.07, p=0.95, Cohen’s d=0.02). These results suggest that a change in topology or diversion of attention to the gap cannot explain our earlier results. Note that a difference in accuracy without a difference in slope cannot be explained simply in terms of speed-accuracy trade-offs. First, speed-accuracy trade-offs result in slower RTs at higher accuracies, but not a change in slope. Second, accuracies were generally very high indicating that the speed-accuracy trade-off, if present, needs to be quite small. Finally, despite the difference in error rates in the two conditions, they are mostly parallel: the error-rate slopes only appear to be shallower in the solid condition due to a floor effect at numerosity 1. That is, the error rates in the two conditions reflect a difference in difficulty but not efficiency. Thus, the most likely explanation of our set of results, including the generalisation in Supplementary Experiment 1, is that parts distributed on more than one object are harder to enumerate than parts of one object. This supports the proposal that subitizing is object based.

# References

Chen, L. (2005). The topological approach to perceptual organization. *Visual Cognition*, *12*(4), 553–637.

Egly, R., Driver, J., & Rafal, R. D. (1994). Shifting visual attention between objects and locations: Evidence from normal and parietal lesion subjects. *Journal of Experimental Psychology: General*, *123*(2), 161.

He, L., Zhou, K., Zhou, T., He, S., & Chen, L. (2015). Topology-defined units in numerosity perception. *Proceedings of the National Academy of Sciences*, *112*(41), E5647–E5655. https://doi.org/10.1073/pnas.1512408112

Kluth, T., & Zetzsche, C. (2016). Numerosity as a topological invariant. *Journal of Vision*, *16*(3), 30–30.

1. Primarily due to the restrictions imposed during the Coronavirus pandemic. We had to recruit and test observers who were willing to take part in an hour-long experiment and had the necessary apparatus (a computer with MATLAB and Psychtoolbox, along with a keyboard that included a number pad). [↑](#footnote-ref-1)
